# Description of *Trypoxylonsicklum* sp. nov. from Vietnam and a key to species in the *Trypoxylonfulvocollare* group (Hymenoptera, Crabronidae)

**DOI:** 10.3897/BDJ.12.e114333

**Published:** 2024-05-21

**Authors:** Phong Huy Pham, Alexander V. Antropov, Hieu Van Nguyen

**Affiliations:** 1 Institute of Ecology and Biological Resources, Vietnam Academy of Science and Technology, 18 Hoang Quoc Viet, Cau Giay, Hanoi, Vietnam Institute of Ecology and Biological Resources, Vietnam Academy of Science and Technology, 18 Hoang Quoc Viet, Cau Giay Hanoi Vietnam; 2 Graduate University of Science and Technology, Vietnam Academy of Science and Technology, Hanoi, Vietnam Graduate University of Science and Technology, Vietnam Academy of Science and Technology Hanoi Vietnam; 3 Zoological Museum of Moscow, Lomonosov State University, Bolshaya Nikitskaya str. 2, Moscow, 125009, Moscow, Russia Zoological Museum of Moscow, Lomonosov State University, Bolshaya Nikitskaya str. 2, Moscow, 125009 Moscow Russia; 4 Faculty of Biology and Agricultural Engineering, Hanoi Pedagogical University 2, 32 Nguyen Van Linh Street, Xuan Hoa, Phuc Yen, Vinh Phuc, Vietnam Faculty of Biology and Agricultural Engineering, Hanoi Pedagogical University 2, 32 Nguyen Van Linh Street, Xuan Hoa, Phuc Yen Vinh Phuc Vietnam

**Keywords:** Crabronidae, new species, taxonomy, *Trypoxylon*, Vietnam

## Abstract

**Background:**

*Trypoxylon* Latreille, 1796 (Hymenoptera, Crabronidae) consists of 633 known species worldwide and the genus is divided into numerous species groups. In Vietnam, 19 species of *Trypoxylon* have been recorded to date. In this study, a new species, *Trypoxylonsicklum* Pham and Antropov sp. nov. is described and illustrated from Vinh Phuc Province, Vietnam. The new species is the second member of the *Trypoxylonfulvocollare* species group. A key to species in the *Trypoxylonfulvocollaris* group is presented.

**New information:**

*Trypoxylonsicklum* Pham and Antropov sp. nov., is described as a new species and is the second member of the *Trypoxylonfulvocollare* species group. A key to species in the *Trypoxylonfulvocollaris* group is presented.

## Introduction

*Trypoxylon* is worldwide in distribution with 633 described species. These wasps are solitary members of the family Crabronidae (Sann et al. 2018, Pulawski 2023). The genus consists of two subgenera, *Trypoxylon* and *Trypargilum*. Whereas the former, consisting of 544 species, is distributed in the Ethiopian, Palearctic, Oriental and Australian Regions, the latter, consisting of 89 species, is distributed only in the Neotropical and Nearctic Regions. Based on external morphological characters and male genitalia structures, *Trypoxylon* is divided into numerous species groups (Richards 1934, Tsuneki 1978a, Tsuneki 1981, Antropov 1994, Antropov 2003, Antropov 2011).

In Vietnam, Pham et al. (2015) and Pham and Antropov (2021) recorded seven *Trypoxylon* species. Recently, Pham et al. (2023) have recorded 14 species in North Vietnam, 12 of them being new records. Altogether, 19 species of the genus have been recorded for the country. During a study course of Hymenoptera in 2023 in Vinh Phuc Province, we collected three specimens of a *Trypoxylon* species. After a closer examination of these specimens, we conclude that they represent a new species and describe it here.

Tsuneki (1981) defined the *Trypoxylonfulvocollare* species group, based on male genitalia characters including the shoulder of the penis valve being almost horizontal; paramere bifurcate at apex; apical lobes of paramere appropriately similar in length; bifurcation deep, reaching top of inner expansion of basiparamere; ventral one of apical lobes of paramere much broader and slightly shorter than dorsal lobe; inner margin of ventral lobe of paramere and that of outer area of basiparamere in a line running straight. The species group previously consisted of a single species, *T.fulvocollare Cameron 1904* (Tsuneki 1978b,Tsuneki 1979, Tsuneki 1981, Pulawski 2023). The new species described here belongs to this species group as it exhibits the above-mentioned characters, thus representing a second species. Lastly, we present a key to the two species of the *Trypoxylonfulvocollare* group.

## Materials and methods

Sampling was carried out using trap nests, which were placed on horizontal branches, 1 - 2.5 m from the ground with their entrances orientated south and north. The trap nests were maintained in the field from 15 to 20 days. Trap nests occupied by *Trypoxylon* were collected and wasps were reared under laboratory conditions. Adult wasps that emerged from these trap nests were kept, euthanised with a killing jar charged with ethyl acetate, pinned and described with the aid of a light stereomicroscope (Nikon SMZ745). Photographic images were taken using a Nikon SMZ800N microscope camera. Morphological terms used in the text follow Bohart and Menke (1976) and Tsuneki (1979).

Specimens examined including the holotype and the paratype of the new species are deposited in the Institute of Ecology and Biological Resources (IEBR), Vietnam Academy of Science and Technology, Ha Noi, Vietnam.

The following abbreviations are used in the text:

- A(*x*): Antennal joint, *x* being the joint number
- ASR: Antennal socket rim
- PAF: Furrow between ASR and SAT
- SAT: Supraantennal tubercle
- G(g): Gastral segment, g being the gastral number.

## Taxon treatments

### Trypoxylon sicklum

Pham & Antropov sp. nov.

9CD7F489-710D-5A49-8E68-AC92874F9CFF

27CFAE77-EF91-44FE-8BFC-DDA9D2C6811B

#### Materials

- **Type status:** Holotype. **Occurrence:** occurrenceID: 4CFA42D6-014D-520D-8674-71C7C28468D8
- **Type status:** Holotype. **Occurrence:** catalogNumber: VP202307050001; recordNumber: 2023; recordedBy: Phong Huy Pham; individualCount: 1; sex: female; lifeStage: adult; occurrenceStatus: Present; disposition: in collection; occurrenceID: C01CD1AE-2EB1-574F-AD84-2933E5988EF7; **Taxon:** scientificName: Hymenoptera, Crabronidae, Trypoxylonsicklum Pham & Antropov, sp. nov.; higherClassification: Insect; kingdom: Animalia; order: Hymenoptera; family: Crabronidae; genus: Trypoxylon; specificEpithet: sicklum; taxonRank: species; scientificNameAuthorship: Pham & Antropov, 2023; **Location:** country: Vietnam; stateProvince: Vinh Phuc; locality: Me Linh Station for Biodiversity, Ngoc Thanh commune, Phuc Yen City; locationRemarks: Vinh Phuc: Me Linh Station for Biodiversity, Ngoc Thanh commune, Phuc Yen City, 5 July 2023, trap nests; georeferenceProtocol: label; **Identification:** identifiedBy: Phong Huy Pham; dateIdentified: 2023; **Event:** samplingProtocol: trap nests; eventDate: 05/07/2023; **Record Level:** language: en; collectionCode: Insects; basisOfRecord: PreservedSpecimen
- **Type status:** Holotype. **Occurrence:** catalogNumber: VP202307050002; recordNumber: 2023; recordedBy: Phong Huy Pham; individualCount: 1; sex: male; lifeStage: adult; occurrenceStatus: Present; disposition: in collection; occurrenceID: E5E0EAC9-0776-5013-96DA-D8A2FBEB32FB; **Taxon:** scientificName: Hymenoptera, Crabronidae, Trypoxylonsicklum Pham & Antropov, sp. nov.; higherClassification: Insect; kingdom: Animalia; order: Hymenoptera; family: Crabronidae; genus: Trypoxylon; specificEpithet: sicklum; taxonRank: species; scientificNameAuthorship: Pham & Antropov, 2023; **Location:** country: Vietnam; stateProvince: Vinh Phuc; locality: Me Linh Station for Biodiversity, Ngoc Thanh commune, Phuc Yen City; locationRemarks: Vinh Phuc: Me Linh Station for Biodiversity, Ngoc Thanh commune, Phuc Yen City, 5 July 2023, trap nests; georeferenceProtocol: label; **Identification:** identifiedBy: Phong Huy Pham; dateIdentified: 2023; **Event:** samplingProtocol: trap nests; eventDate: 05/07/2023; **Record Level:** language: en; collectionCode: Insects; basisOfRecord: PreservedSpecimen
- **Type status:** Paratype. **Occurrence:** catalogNumber: VP202307050003; recordNumber: 2023; recordedBy: Phong Huy Pham; individualCount: 1; sex: male; lifeStage: adult; occurrenceStatus: Present; disposition: in collection; occurrenceID: 1F767E5C-E000-55DC-B815-FF5E4C076137; **Taxon:** scientificName: Hymenoptera, Crabronidae, Trypoxylonsicklum Pham & Antropov, sp. nov.; higherClassification: Insect; kingdom: Animalia; order: Hymenoptera; family: Crabronidae; genus: Trypoxylon; specificEpithet: sicklum; taxonRank: species; scientificNameAuthorship: Pham & Antropov, 2023; **Location:** country: Vietnam; stateProvince: Vinh Phuc; locality: Me Linh Station for Biodiversity, Ngoc Thanh commune, Phuc Yen City; locationRemarks: Vinh Phuc: Me Linh Station for Biodiversity, Ngoc Thanh commune, Phuc Yen City, 5 July 2023, trap nests; georeferenceProtocol: label; **Identification:** identifiedBy: Phong Huy Pham; dateIdentified: 2023; **Event:** samplingProtocol: trap nests; eventDate: 05/07/2023; **Record Level:** language: en; collectionCode: Insects; basisOfRecord: PreservedSpecimen

#### Description

**Female**. Body length 14.2 mm, forewing length 10.3 mm.

Colour. Body black with following parts yellow: A1–A4; base of A5; band immediately after apical margin of clypeus; base of mandible; maxillary and labial palps; tegula; pronotal collar; pronotal lobe; fore coxa; base of mid- and hind-coxae; fore- and mid-trochanters ventrally, femora, tibiae, and tarsi; half of base of hind tibia; lateral sides of petiole, broad base of G2 and G3; apical bands of G1–G5. Following parts ferruginous: apical margin of clypeus; apical two-thirds of mandible; two-thirds of apex of A5, A6–A12; hind trochanter, femur, half apex of tibia and tarsi; half base of petiole dorsally; sides and ventral surface of G6. Veins of wings yellow to brown.

Vestiture. Golden on head and mesosoma, white on metasoma.

**Figure 1.**
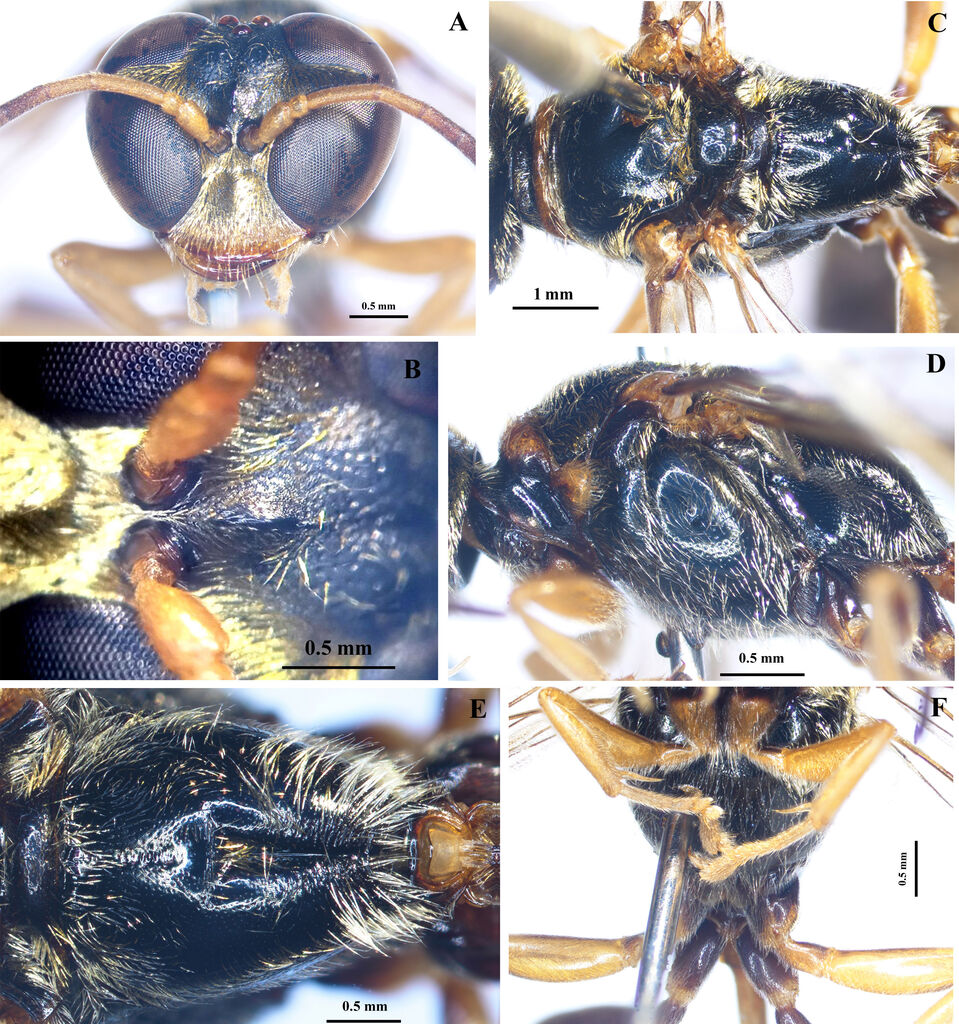
*Trypoxylonsicklum* Pham & Antropov sp. nov., holotype, female. **A** head, frontal view; **B** frons; **C** mesosoma, dorsal view; **D** mesosoma, lateral view; **E** propodeum, dorsal view; **F** mesosoma, ventral view.

Head (Fig. 1A, B). In anterior view, nearly round, width about 1.2× height, its sides roundly convergent below; apex of clypeus blade-shaped, round apically; mandible without tooth; SAT moderately raised with short median carina; ASR moderately high with three distinct carinae; PAF shallow, flat-bottomed; ocelli round and distinctly protruded; frons with rather narrow furrow medially and small punctures, sparse on median area and dense on lateral sides; vertex depressed below level of upper eye margin, with small sparse punctures; occipital carina complete; relative lengths of A1: A2: A3: A4: A5 = 27: 14: 48: 28: 28, respectively; relative lengths of minimum interocular distance at the vertex: Ocellocular distance (distance between inner margin of eye and outer margin of hind ocellus): Ocellar diameter: Postocellar distance (distance between inner margins of hind ocelli) = 37: 3: 12: 7, respectively; ratio of minimum interocular distance at the vertex: minimum interocular distance at base of clypeus = 1.

**Figure 2.**
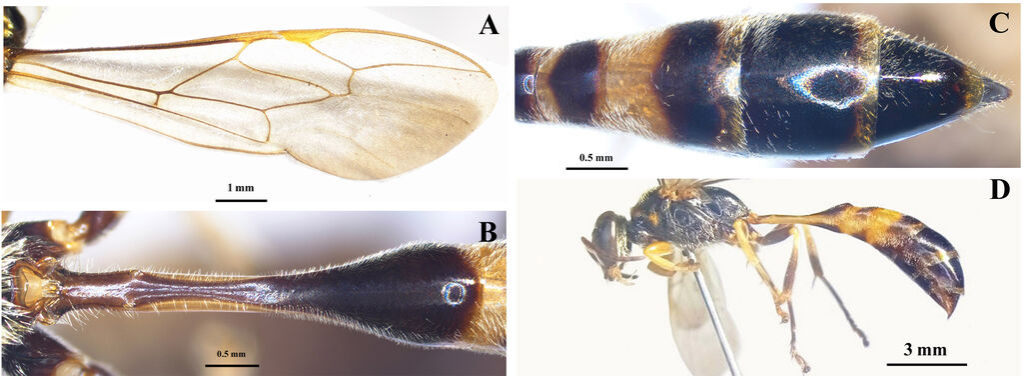
*Trypoxylonsicklum* Pham & Antropov sp. nov., holotype, female: **A** fore wing; **B** petiole; **C** metasoma, dorsal view; **D** habitus, lateral view.

Mesosoma (Fig. 1C-F, Fig. 2A). Pronotal collar with anterior part moderately swollen posteromedially, posterior part discoloured; pronotal lamella triangularly protruded, with apical angle approximately 150°; subalar area of mesopleuron with pent-roofed structure distinctly developed; mesoscutum, scutellum, metanotum, propodeum with sparse, fine, small punctures; mesopleuron with rather dense punctures; propodeum without lateral carina, with median groove dorsally, shallow on basal half and deep on apical half, posterior surface with a series of horizontal striae and median grooves conspicuously deep; gastral socket rim simple, nearly roundly elevated. Wings hyaline; forewing with stigma as long as prestigma.

Metasoma (Fig. 2B-D). Shiny, with very sparse micropunctures on petiole and G2–G5; petiole flask-shaped, length about 4× its apical width and about 1.3× that of G2 and G3 combined, with its apex more highly protruding than base of G2; tergum of G6 rounded apically, rugose basally, impunctate, its apex slightly curved upwards.

**Male**. Body length 12.6 mm, forewing length 9.1 mm.

Structures as in female, but differing as follows:

Colour. A1–A7 yellow; A8–A13, half apex of clypeus, maxillary and labial palps ferruginous, half apex of mandible black.

**Figure 3.**
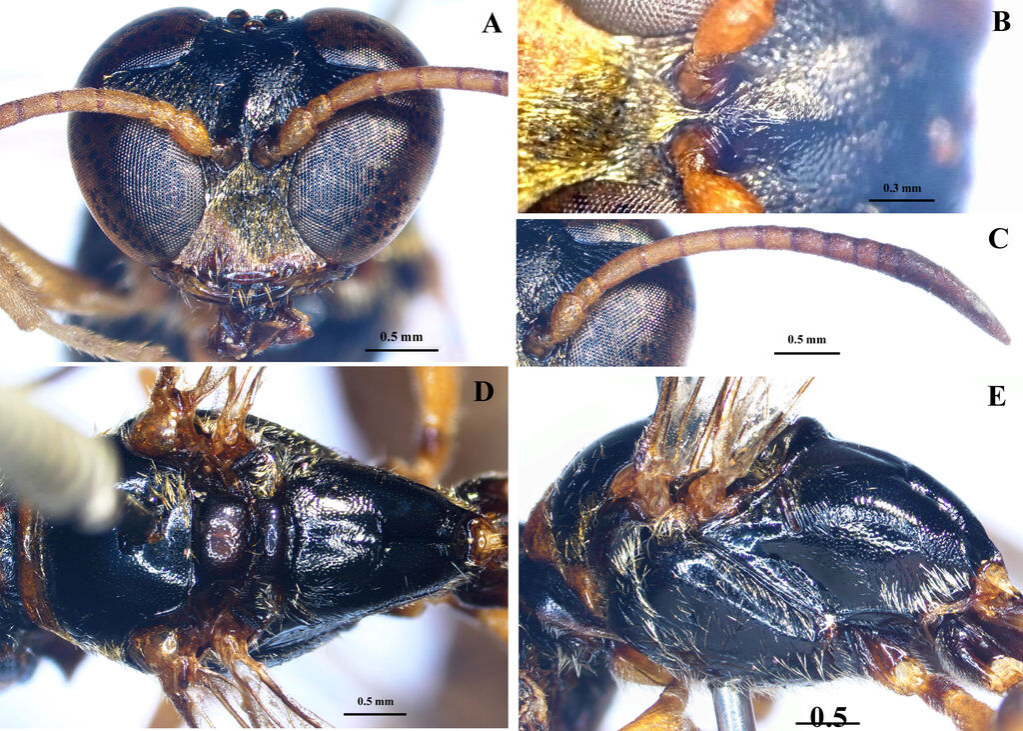
*Trypoxylonsicklum* Pham & Antropov sp. nov., paratype, male. **A** head, frontal view; **B** frons; **C** antenna; **D** mesosoma, dorsal view; **E** mesosoma, lateral view.

Head (Fig. 3A-C). A3 shorter than A1 and A2 combined, but longer than A4, A5; relative length of A1: A2: A3: A4: A5 = 19: 9: 20: 15: 15; A13 as long as A9–A12 combined; relative length of minimum interocular distance at the vertex: Ocellocular distance: Ocellar diameter: Postocellar distance = 32: 5: 8: 6, respectively.

**Figure 4.**
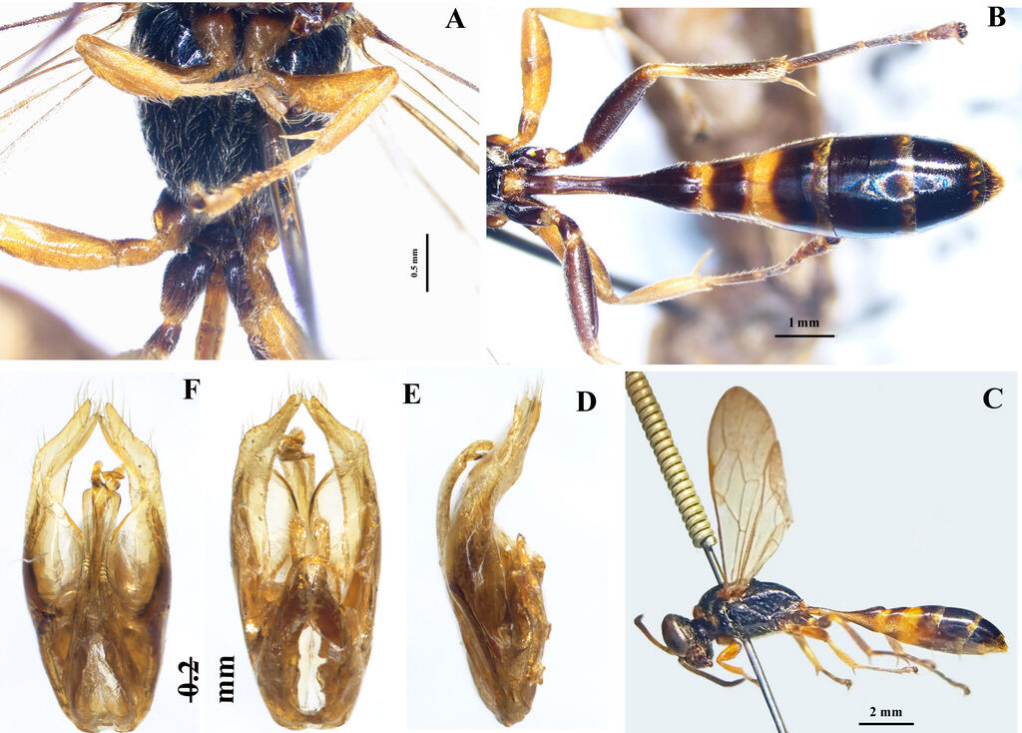
*Trypoxylonsicklum* Pham & Antropov sp. nov., paratype, male and genitalia. **A** mesosoma, ventral view; **B** metasoma, dorsal view; **C** habitus, lateral view; **D** genitalia, lateral view; **E** genitalia, ventral view; **F** genitalia, dorsal view.

Metasoma (Fig. 4A-C). Petiole with length 3.5× its apical width.

Genitalia (Fig. 4D-F). Brownish-yellow, except apical part of sickle black. Penis valve with shoulder, sickle and apical part; shoulder horizontal; sickle conspicuously curved, with its apex distinctly pointed; apical part moderately curved; paramere with two lobes at apex (namely, ventral and dorsal lobes), their length about equal, ventral lobe much broader than dorsal lobe; furrow between two lobes deep, reaching apex of inner expansion of basiparamere; inner margin of ventral lobe and inner margin of outer area of basiparamere in a line moderately curved medially. Dorsal lobe with some long, erect setae apically; ventral lobe with 5–6 long, erect setae at apex, subapical part with several sparse, erect setae along outer margin.

#### Diagnosis

The new species runs close to *T.shakha* Tsuneki, 1979 in a key of*Trypoxylon* of the Indian subcontinent and Southeast Asia (Tsuneki 1979). In *T.shakha* and in the newly-described species, the females display the following characters: vertex depressed below level of upper eye margin; PAF flat-bottomed; occipital carina complete; propodeum without lateral carinae, dorsal area with furrow; frons, mesoscutum, scutellum, metanotum and propodeum with small sparse punctures. Following parts with yellow integument: A1–A4, pronotal collar, fore- and mid-legs, except broad base of mid-coxa, tergal base of G2–G4, apical bands of G3–G5. Vestiture golden on head and mesosoma. Females of the new species differ from those of *T.shakha* as follows: Clypeus round apically (in *T.shakha*, clypeus not round apically, with median margin highly protruded and truncated apically); PAF shallow (in *T.shakha*, PAF deep); SAT dorsally with medial carina short, not enlarged, not broadly excavated (in *T.shakha*, SAT dorsally with median carina enlarged into a smooth and round area and broadly excavated); ASR moderately raised (in *T.shakha*, ASR highly raised, bicarinate on apex); vestiture white on metasoma (in *T.shakha*, vestiture golden on metasoma).

#### Etymology

The specific name of this new species refers to the sickle shape of the penis valve.

#### Distribution

Vietnam: Vinh Phuc Province.

## Identification Keys

### Key to species in the *Trypoxylonfulvocollare* group, females

**Table:** 

1	Vertex depressed much below level of upper eye margins; distance between inner margins of hind ocelli equal to distance between inner margin of eye and outer margin of hind ocellus; propodeum with lateral carinae, dorsal area without lateral furrows; petiole as long as G2 and G3 combined	*T.fulvocollare* Cameron, 1904
–	Vertex depressed moderately below level of upper eye margins; distance between inner margins of hind ocelli 2× distance between inner margin of eye and outer margin of hind ocellus; propodeum without lateral carinae, dorsal area with lateral furrows; petiole much longer than G2 and G3 combined	*T.sicklum* sp. nov. Pham & Antropov

### Key to species in the *Trypoxylonfulvocollare* group, males

**Table:** 

1	A13 as long as A8–A12 combined; petiole with length 4-5× its apical width; subapical part of ventral lobe of paramere with rather dense, erect setae; sickle brownish-yellow, lightly to moderately curved; inner margin of ventral lobe and that of outer area of basiparamere in a line straight or slightly curved medially	*T.fulvocollare* Cameron, 1904
–	A13 shorter than A8–A12 combined; petiole with length 3.5× its apical width; subapical part of ventral lobe of paramere with some long, erect setae; sickle conspicuously curved, with apical part black; inner margin of ventral lobe and that of outer area of basiparamere in a line moderately curved medially	*T.sicklum* sp. nov. Pham & Antropov

## Discussion

As the male of *T.shaka* is unknown, it has not been placed in any *Trypoxylon* species group. External morphological characters of females of *T.shakha* are similar to those of *T.sicklum* sp. nov., suggesting that *T.shakha* may be a member of the *T.fulvocollare* species group. To clear this matter, future morphological studies on males of *T.shaka* are required.

*Trypoxylonsicklum* sp. nov. was found in a montane forest habitat, about 350 m in elevation, in Vinh Phuc Province of Vietnam. The wasp used two trap nests for its nesting site. Most *Trypoxylon* species construct their nests in pre-existing cavities, such as holes in wood, bamboo and other plant stems and in abandoned mud nests of other wasps. Use of trap nests is common in *Trypoxylon* and has been reported for various species, such as *T.carinatum*, *T.frigidum*, *T.kolazyi*, *T.clavatum*, *T.collinum*, *T.lactitarse* and *T.tridentatum* (see Krombein (1967)); *T.deceptorium*, *T.clavicerum*, *T.figulus* and *T.scutatum* (see Kazenas (2001)); and *T.bicolor* (see Vicens et al. (2022)). In contrast, several species in the genus (the subgenusTrypargilum) construct their nests with mud and are, thus, called pipe-organ wasps, for example *T.monteverdeae* (see Brockmann (1992)) and *T.politum* (see Brockmann and Grafen (1992)).

## Supplementary Material

XML Treatment for Trypoxylon sicklum
